# Eyelid Edema: A Rare Cause of a Common Sign

**DOI:** 10.1155/2017/9193706

**Published:** 2017-08-07

**Authors:** Andreia Soares, Cristina Almeida, Cristina Freitas, Marco Sales-Sanz, Sara Ribeiro

**Affiliations:** ^1^Hospital de Braga, School of Medicine, University of Minho, Braga, Portugal; ^2^Ophthalmology Department, Hospital Universitario Ramón y Cajal, Madrid, Spain; ^3^Ophthalmology Department, Hospital Lusíadas, Porto, Portugal

## Abstract

We report a 48-year-old female patient who presented to the emergency room with right eyelid edema, with 3 days of evolution. She had suffered minor trauma to this eye one week before. She reported episodes of right eyelid swelling of spontaneous resolution since the occurrence of a traumatic brain injury 5 years ago. Ophthalmological examination showed a soft and painless eyelid edema of the right eye. Brain computed tomography showed an area of bone discontinuity of the orbital roof with brain herniation and a CSF leak into the eyelid (blepharocele). Magnetic resonance confirmed the result of TC and revealed an area of frontal encephalomalacia. Ibuprofen (800 mg/day) was prescribed, with complete resolution within 20 days. She was evaluated by Neurosurgery with no indication of surgery due to the resolution of the edema and absence of symptoms. Blepharocele is a rare entity that should be considered in the differential diagnosis of unilateral eyelid edema. It can be secondary to an orbital fracture or congenital lesion.

## 1. Introduction

A cerebrospinal fluid fistula is defined as the communication between the subarachnoid space and the external environment [1]. These fistulas complicate 2% of all head traumas and occur in 12–30% of all basilar skull fractures. Otorrhea or rhinorrhea are more common presentations, but, in rare situations, CSF can collect in the orbit (orbitocele) or in the upper eyelid (blepharocele) [2]. Most of the few cases described in the last 55 years have occurred in children, probably due to the immaturity of a child's frontal sinus [3–8]. Ommaya et al. [9] proposed a classification of cerebrospinal fluid fistulas, dividing them into traumatic (iatrogenic or accidental) or nontraumatic origin (idiopathic or secondary).

Blepharocele is a rare entity. If the content is only CSF, the eyelid swelling may be transilluminant. The onset of the fistulas may be seen later in the form of eyelid swelling precipitated by a microtrauma, independently of their etiology (congenital or traumatic) [1, 3, 10].

We report here the case of a female adult patient with blepharocele precipitated by a microtrauma.

## 2. Case Report

**Figure 1:**
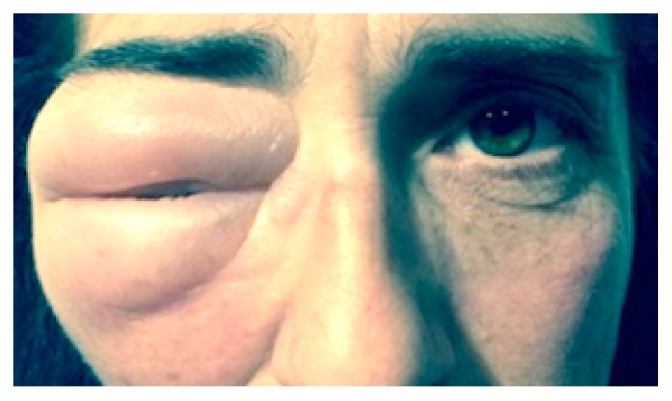
Clinical appearance of the patient with right eyelid edema.

A 48-year-old female patient presented in the emergency room with eyelid edema in the right eye, with 3 days of evolution (Figure 1). She had suffered a minor trauma to this eye one week before, caused by the hand of her 2-year-old son. She reported previous episodes of swelling in the same eye, with spontaneous improvement, since the occurrence of a major head trauma five years ago. At that time, she had not been evaluated by a medical professional and had not been submitted to any neuroradiological exam.

**Figure 2:**
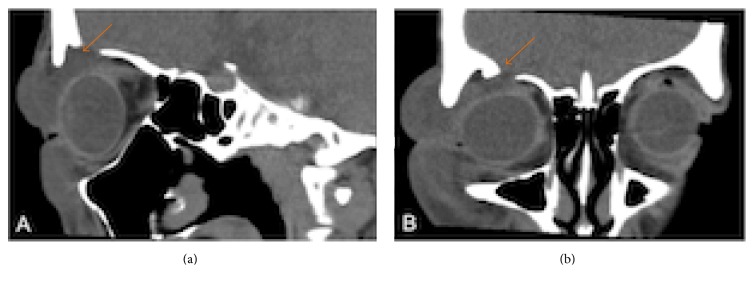
Orbital CT scans ((a) sagittal scan, (b) coronal scan) revealed an area of bone discontinuity in the right orbital roof with adjacent cephalocele (arrow).

**Figure 3:**
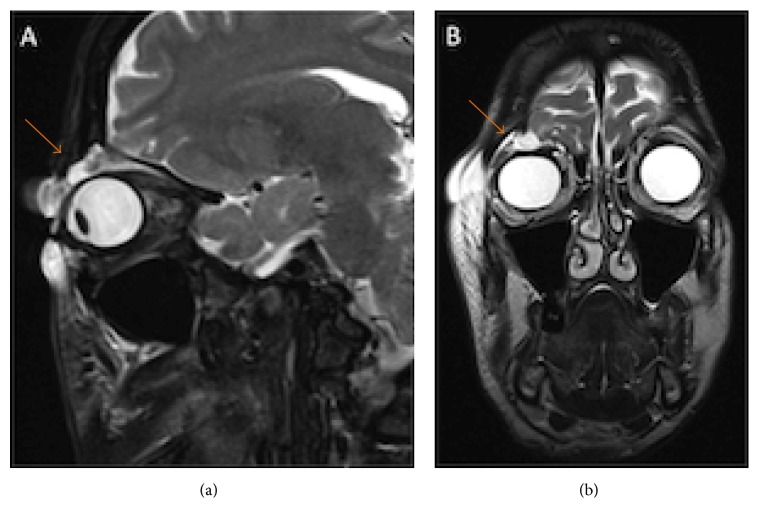
Orbital MR ((a) sagittal scan, (b) coronal scan) showed an area of bone discontinuity in the anterior right orbital roof with adjacent cephalocele (extraconic) and a frontobasal area of encephalomalacia related to trauma.

The ophthalmological examination showed a soft and painless eyelid edema of the right eye, without signs of inflammation or cutaneous lesions. Best corrected visual acuity was 20/20 in both eyes. Pupils were equal and reactive to light. Ocular motility, exophthalmometry, anterior biomicroscopy, intraocular pressure, and fundoscopy were normal. Brain tomography scans showed an area of bone discontinuity of the orbital roof with brain herniation and a CSF leak into the eyelid (Figure 2). Magnetic resonance of the orbits confirmed the result of computed tomography (CT) and revealed an area of frontal encephalomalacia probably related to the previous trauma (Figure 3). She was treated with a nonsteroidal anti-inflammatory drug (ibuprofen, 800 mg/day) for 2 weeks, with total improvement of the edema. Neurosurgical evaluation was requested and there was no indication for surgery due to resolution of the edema and absence of symptoms. Neurological examination was normal, with no focal deficits.

## 3. Discussion

Blepharocele is a rare ophthalmological entity. The main cause is head trauma, but the condition can be associated with congenital lesions. We found 9 cases described in the literature (Table 1), 8 with traumatic etiology and one with congenital etiology [1, 3–5, 7, 8, 10–12]. The etiology of the blepharocele of our patient is undetermined. It could be associated with the head trauma she had 5 years ago, as suspected by the area of encephalomalacia, probably related to a cerebral contusion. However, we have no records of a clinical evaluation or any neuroradiological exam of this event or of previous brain and orbit imaging exams.

CSF leaks have been reported to manifest months or even years after the initial trauma. The onset of the fistulas may be seen in the form of eyelid swelling late after the trauma. The literature contains few reports of CSF leakage whose onset was delayed by more than 30 years [12]. Several precipitating factors such as coughing and undetected minor traumas could be responsible for this unusually late manifestation of the fistula. In our case, it could be associated with the recent minor trauma caused by the hand of her son. The temporary seal provided by a clot, inflammatory granulation, contusional cerebral adhesions, or mucocele may be broken down, resulting in late accumulation [12].

On the other hand, this late manifestation may be associated with a congenital lesion. Ommaya et al. [9] reported a pathophysiological explanation for nontraumatic blepharocele. Their theory, called focal atrophy, is based on a reduction of volume of cribriform structures and the sella turcica by an ischemic mechanism. The space created would be filled with CSF, exerting an erosive force in the skull. Bone defects in the skull base allow the creation of small meningeal hernias which lead to the formation of cerebrospinal fluid fistula [1].

Most traumatic CSF leaks resolve spontaneously without treatment, the majority within the first 24–48 hours, as a result of blood products and/or inflammatory adhesions at the site of the dural breach and associated skull fracture. Herniation of the brain tissue into the traumatic defect may also play a role in the cessation of the leak. Aspiration of posttraumatic eyelid swelling may result in disastrous consequences and should not be done. Patients with leaks that persist for more than 24 hours may be at increased risk for meningitis, and surgical intervention is required. The cosmetic and functional results of surgery have been reported to be excellent [3, 4, 10].

Blepharocele is a rare condition but it is important to consider this diagnosis in patients with eyelid swelling without resolution after minor or major traumas.

## Figures and Tables

**Table 1:** Literature review of blepharocele case reports.

	Author, year	Gender/age	Mechanism	Clinical manifestation	Treatment
(1)	Bagolini, 1957	NS/9 m	Car accident	Tearing (“Oculorrhea”) UE Hematoma Anisocoria	Surgical

(2)	Garza-Mercado, 1982	M/20 y	Assault	Tearing (“Oculorrhea”) Eyelid edema Ecchymosis Limited EOM	Surgical

(3)	Till, 1987	M/14 m	Stabbing wound	Tearing (“Oculorrhea”) Eyelid edema	Surgical

(4)	Bhatoe, 2002	M/25 y	Traffic collision	Eyelid swelling Periorbital ecchymosis	TC surgery Dural repair

(5)	Arslantas, 2003	NS/3 y	Fall	Orbitocele	TC surgery Dural repair

(6)	Chandra, 2013	F/4 y	Blunt head injury	UE swelling	TC surgery Dural repair

(7)	Borumandi, 2013	F/49 y	Fall	Eyelid swelling Ecchymosis	Conservative

(8)	Govindaraju, 2013	M/43 y	Head injury	Eyelid swelling	TC surgery Dural repair

(9)	Germano, 2015	F/51 y	Congenital lesion	Eyelid swelling	TC surgery Dural repair

y: years; m: months; NS: not stated; EOM: extraocular movements; UE: upper eyelid.
